# Reconsidering the Avian Nature of the Oviraptorosaur Brain (Dinosauria: Theropoda)

**DOI:** 10.1371/journal.pone.0113559

**Published:** 2014-12-10

**Authors:** Amy M. Balanoff, G. S. Bever, Mark A. Norell

**Affiliations:** 1 Stony Brook University, Department of Anatomical Sciences, Stony Brook, New York, United States of America; 2 New York Institute of Technology, College of Osteopathic Medicine, Department of Anatomy, Northern Boulevard, Old Westbury, New York, United States of America; 3 American Museum of Natural History, Division of Paleontology, Central Park West at 79^th^ Street, New York, New York, United States of America; University of Lethbridge, Canada

## Abstract

The high degree of encephalization characterizing modern birds is the product of a long evolutionary history, our understanding of which is still largely in its infancy. Here we provide a redescription of the endocranial space of the oviraptorosaurian dinosaur *Conchoraptor gracilis* with the goal of assessing the hypothesis that it shares uniquely derived endocranial characters with crown-group avians. The existence of such features has implications for the transformational history of avian neuroanatomy and suggests that the oviraptorosaur radiation is a product of the immediate stem lineage of birds—after the divergence of *Archaeopteryx lithographica*. Results derived from an expanded comparative sample indicate that the strong endocranial similarity between *Conchoraptor* and modern birds largely reflects shared conservation of plesiomorphic features. The few characters that are maintained as being uniquely expressed in these two taxa are more likely products of convergence than homology but still indicate that the oviraptorosaur endocranial cavity has much to teach us about the complex history of avian brain evolution.

## Introduction

Oviraptorosauria is a monophyletic group of morphologically peculiar theropod dinosaurs from the Cretaceous of North America and Asia. The phylogenetic relationship of these dinosaurs to the origin of crown-group birds, Aves *sensu* Gauthier 1986 [1], and their immediate stem lineage (Avialae) is a point of enduring controversy. Derived oviraptorosaurs exhibit an array of bird-like features, prompting the suggestion that they are secondarily flightless avialans more closely related to Aves than is *Archaeopteryx lithographica* [2]–[4]. The most recent and comprehensive phylogenetic analyses reject this hypothesis, instead supporting an oviraptorosaur divergence near the base of the more inclusive Maniraptora—a position where their bird-like features are more parsimoniously explained as convergence than homology (e.g., [5]–[9]).

The evolutionary origin of the highly encephalized avian brain as revealed by the endocranial morphology of non-avian theropod dinosaurs continues to be a popular research topic of wide-ranging interest [10]–[20]. Perhaps surprisingly, details of the oviraptorosaur endocranial space have yet to be integrated into this general transformational model. The first, and still one of the only, oviraptorosaur endocasts to be thoroughly described is that of *Conchoraptor gracilis* [21], [22]. This endocast was characterized as possessing features that were otherwise considered exclusively avialan and typically associated with the origin of avian powered flight. These include an encephalization index (endocranial volume relative to estimated body mass) that falls solidly within the range of extant birds and outside that of the then-sampled non-avian dinosaurs. The recognition of such derived, avian-like neuroanatomical features, and the broader phylogenetic conclusions drawn from them, are congruent with those of an earlier study that used vascular scars on the internal surface of a fragmentary oviraptorid frontoparietal to argue for the presence of a highly encephalized forebrain and for the avialan status of the group as a whole [23].

Our study uses a previously undescribed specimen of *Conchoraptor gracilis* to build on the work of Kundrát (2007) [21] and Kundrát and Janácek (2007) [22]. The preservational quality of the specimen, in combination with a taxonomically expanded dataset, provides new insight into the anatomical details of the endocranial space of *Conchoraptor* and facilitates a critical reassessment of the implications of these details for the deep history of avian neuroanatomy and the taxonomic status of oviraptorosaurs.

## Methods

**Figure 1:**
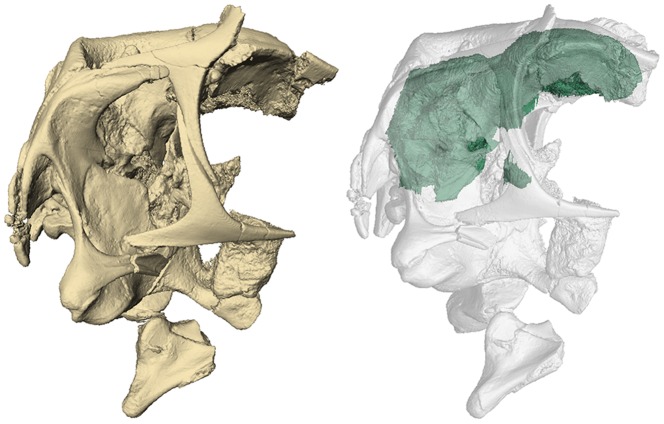
Three-dimensional rendering of the braincase of *Conchoraptor gracilis* (IGM 100/3006). The left image depicts the fully rendered braincase, whereas in the right image the braincase is rendered semi-transparent and the endocranial volume (endocast) is rendered opaque.

We studied the endocranial morphology of the oviraptorid *Conchoraptor gracilis* (IGM [Geological Institute of Mongolia, Ulaan Baatar] 100/3006; Fig. 1) using high-resolution X-ray computed tomography (HRCT) and a comparative sample of avian and non-avian theropods, including the early oviraptorosaur *Incisivosaurus gauthieri* (see [20] for a complete list of examined specimens and scanning parameters). Digital casts of the endocranial cavity (endocasts) were constructed using VGStudioMax 2.1, which was also utilized for all linear and volumetric measurements of the endocranial space.

As in crown birds [24], the brain of non-avian maniraptorans [25] (and independently mammals), including oviraptorosaurs, fills the majority of the cranial cavity and leaves an impression on the deep surface of the surrounding bones, supporting the conclusion that endocasts of these taxa are reasonable estimates of linear, volumetric and geometric proportions of the brain [25]. IGM 100/3006, unlike the specimen of *Conchoraptor* described by Kundrát (2007) [21], ZPAL MgD-I/95 (Institute of Paleobiology, Polish Academy of Science), preserves a complete occipital plate and thus is more complete. A small amount of distortion is present in IGM 100/3006, with the left side displaced slightly rostrodorsally. As an attempt to bypass these effects, we reconstructed the undistorted right half of the endocast and then mirrored it along the median sagittal plane. The presumption is that this approach provides a more accurate estimate of brain morphology as it was during life assuming that the brain is symmetric. Cranial nerves and blood vessels were truncated proximally to minimize their influence on volumetric estimates. Although the cast of each anatomical structure and not the structure itself is being described, for ease of communication, endocast features are referred to by the names of the soft tissues they reflect.

The relationship between body mass and total endocranial volume is assessed using bivariate regression analyses. Body size estimates for all taxa are based on femur length and calculated using the equation of Christiansen and Fariña (2004) [26]. Although no one metric accurately estimates body mass across all groups [27], we utilize a single proxy to maintain consistency throughout the analysis. These data were log transformed to accommodate them onto a single chart and facilitate pattern recognition. Best-fit lines were mapped onto the data using reduced major axis regression. These lines were fit to the crown-avian data points as well as to the paraphyletic group “non-avian theropods” to approximate the “ancestral” condition. These data were tested previously [20] for non-independence caused by phylogenetic influence [28].

## Results

**Figure 2:**
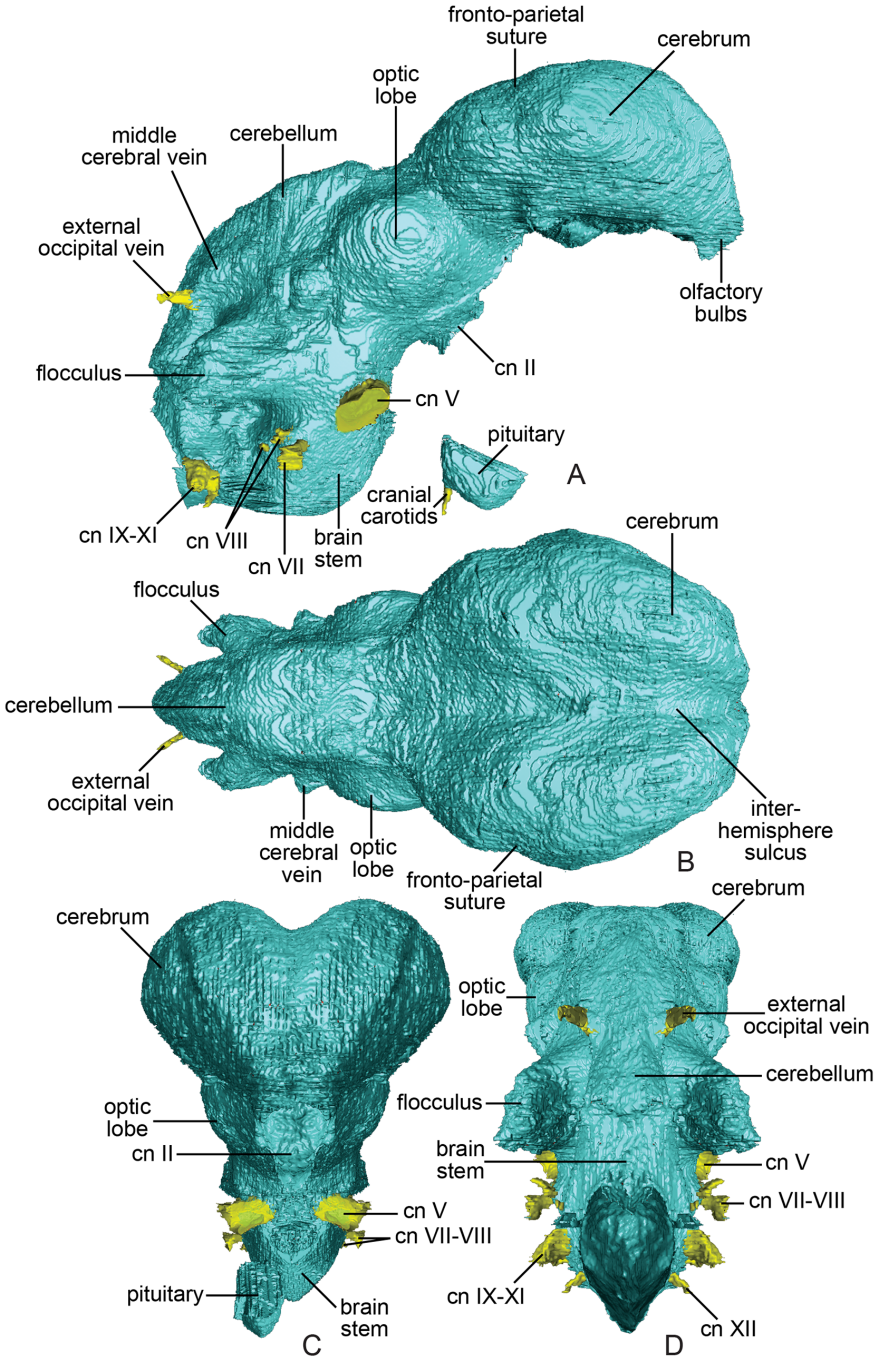
Endocast of *Conchoraptor gracilis* (IGM 100/3006) in (A) right lateral; (B), dorsal; (C), rostral; and (D), caudal views.

The cranial endocast of IGM 100/3006 is mediolaterally wide and, especially in the forebrain region, rounded in shape (Figs. 1, 2). Its overall volume is 9.44 cm^3^. Two distinct points of flexure are present along the long axis of the brain—one immediately caudal to the cerebrum and one within the medulla oblongata [12].

Visible regions of the forebrain include the olfactory bulbs, cerebral hemispheres, and distal end of the pituitary body (Fig. 2). The olfactory tracts are not visible and were likely highly retracted so that they contact the caudal surface of the cerebrum. The olfactory bulbs are small, and the rostral-most end of the bulbs may be truncated due to poor preservation. Despite this truncation, it is unlikely that they constitute more than 0.5% of total endocranial volume. The cerebral hemispheres form the broadest portion of the endocast and are separated by a wide but shallow sagittal intercerebral fissure. The hemispheres taper rostrally, and their shape is more oval than pyriform in dorsal view. The height of the cerebrum in lateral view is relatively consistent along its rostrocaudal length. The pituitary body (hypophysis cerebri) is present as an elongate, rectangular structure that constitutes approximately 0.61% of the total endocranial volume. The infundibular stalk, which would have connected the pituitary to the main body of the diencephalon, is not present in the reconstruction, reflecting poor ossification of the surrounding basisphenoid. The ventrally situated internal carotid canals enter the sella turcica separately and, though they converge towards the midline, fail to anastomose.

The optic tracts and lobes (CN II) are the only discernible features of the diencephalon and midbrain, respectively (Fig. 2). The lobes are positioned directly caudal to the cerebral hemispheres and rostral to the middle cerebral vein. Rather than contacting each other along the sagittal midline, the optic lobes exhibit considerable lateral displacement.

**Figure 3:**
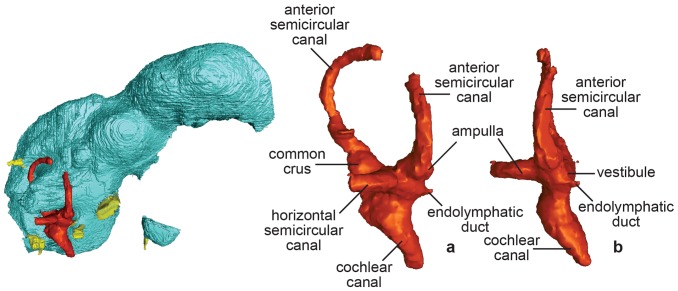
Endocast of the osseous labyrinth of *Conchoraptor gracilis* (IGM 100/3006) in (A), lateral and (B), rostral views.

The visible structures of the hindbrain include the cerebellum, medulla oblongata, and several cranial nerves (CN V-VII, VIII-XI). The trigeminal, facial, and vestibulocochlear nerves exit through separate foramina, whereas the glossopharyngeal, vagus, and accessory nerves presumably exit through the divided metotic fissure along with the jugular vein (Fig. 2). The cerebellum lacks a distinct dural peak but expands dorsally, rostrally, and laterally, nearly reaching the lateral extent of the optic lobes when viewed dorsally. A low occipital sinus runs along the cerebellar midline. There is no evidence of cerebellar folds. The middle cerebral vein (caudal petrosal sinus) is visible on the lateral surface of the cerebellum extending in an arc dorsal to the floccular lobe (Figs. 2–3). The floccular lobe itself (cerebellar auricle) projects caudolaterally from the lateral hindbrain surface (Fig. 2). The medulla oblongata is mediolaterally narrow and demonstrates the aforementioned deep flexure. The divided metotic fissure and internal acoustic fossa are visible as extensions of this region (Fig. 2).

The bony ear labyrinth and cochlear canal of *Conchoraptor gracilis* were previously unknown, and we were unable to produce an endocast for the entire bony labyrinth. Sediment infilling much of the semicircular canals makes them indistinguishable from bone. The visible structures include the majority of the rostral semicircular canal, approximately half of the horizontal canal, the vestibule, and cochlear canal (Fig. 3; Table 1). The rostral semicircular canal is kidney shaped in lateral view, with the apex directed somewhat caudally and extending back to the level of the common crus and caudal semicircular canal. Although the caudal semicircular canal is not visible in IGM 100/3006, the common crus shared between it and the rostral canal is present and extends into the vestibule. A small protuberance on the caudal surface of the caudal arc of the rostral semicircular canal likely marks the divergence of the caudal semicircular canal. This divergence point indicates that the caudal canal was approximately half as tall as the rostral canal and directed laterally. This configuration places the three semicircular canals at approximately orthogonal angles to one another (Table 1). The rostral portion of the horizontal semicircular canal is represented only by a small arc extending laterally from the vestibular surface. The vestibule does not extend dorsal to this canal. The cochlear canal is directed ventromedially towards the midline.

**Table 1:** Select cranial measurements (mm) of IGM 100/3006 and MgD-I/95.

IGM 100/36:		
	Braincase length (occiput to midpoint of orbit)	45.0
	Braincase width (widest point)	33.0
	Cochlear length	7.4
	Cochlear canal diameter 1	2.5
	Cochlear canal diameter 2	1.8
	Rostral semicircular canal length	23.5
	Rostral semicircular canal diameter 1	0.63
	Rostral semicircular canal diameter 2	0.79
	Angle between rostral and horizontal semicircular canals	85°
MgD-I/95:		
	Braincase length	43.0
	Braincase width (widest point)	32.0

## Discussion

**Figure 4:**
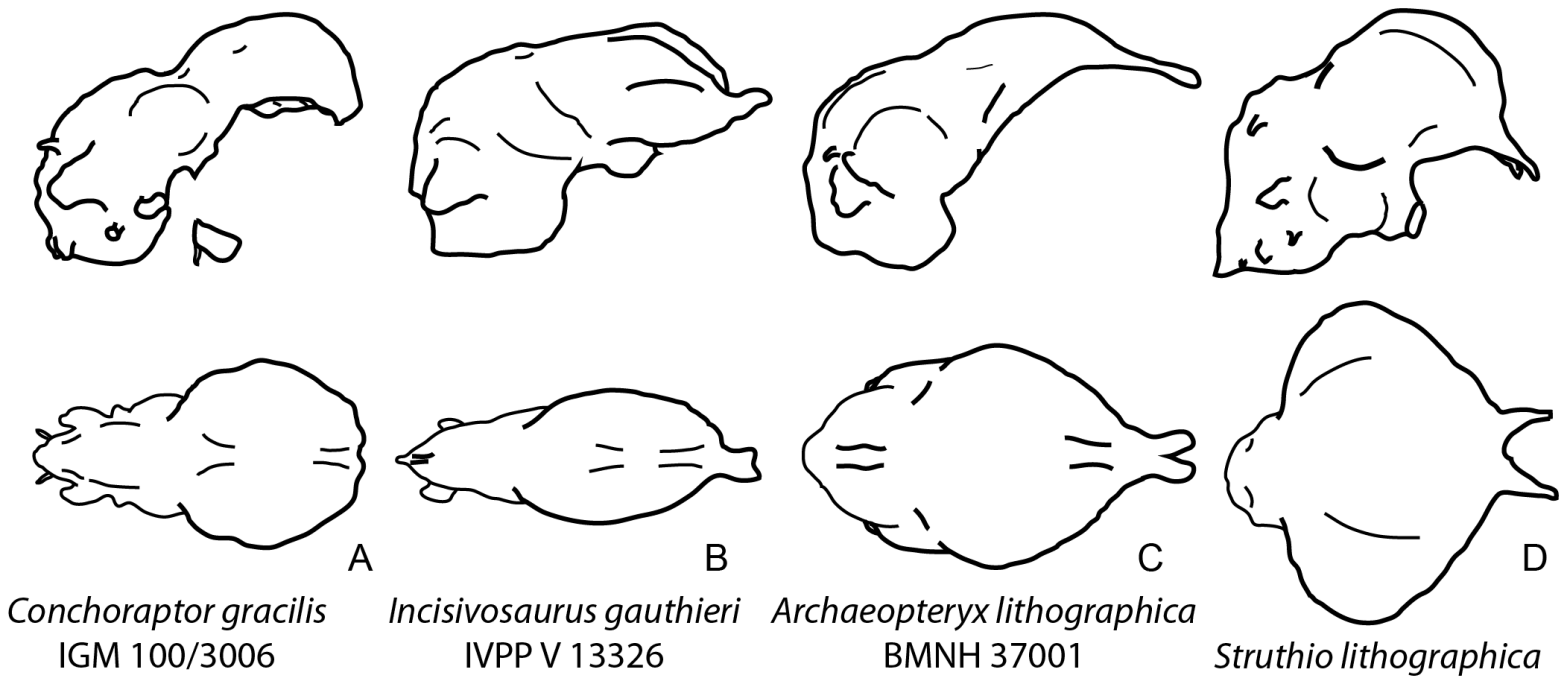
Comparative sample of maniraptoran endocasts. Outlines of endocasts of (**A**), *Conchoraptor gracilis* (IGM 100/3006); (**B**), *Incisivosaurus gauthieri* (IVPP V 13326); (**C**), *Archaeopteryx lithographica* (BMNH 37001); and (**D**), ostrich (*Struthio camelus*). Right lateral view depicted in upper row and dorsal view in lower row.

**Figure 5:**
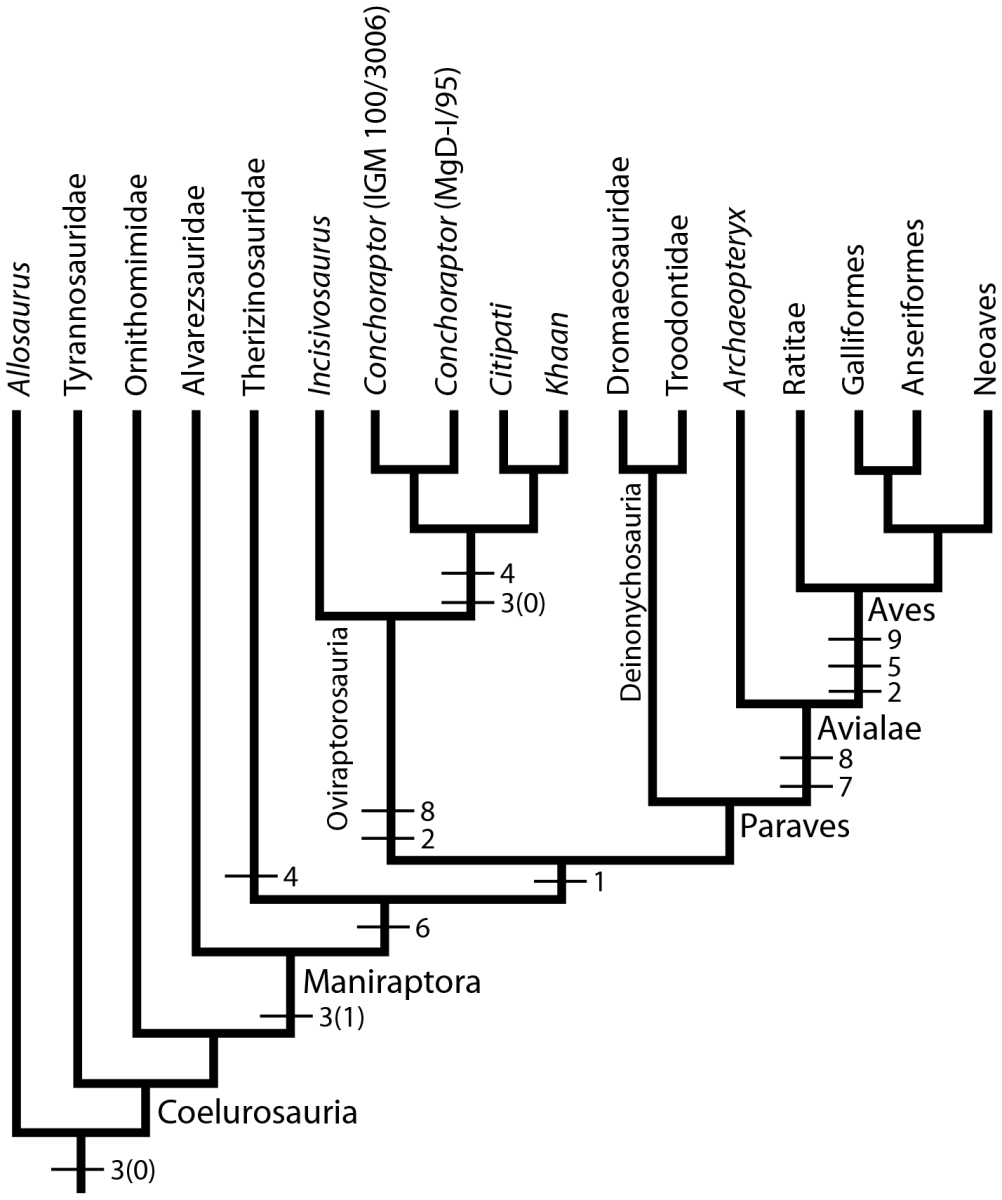
Phylogenetic relationships of coelurosaurian dinosaurs [8]. Tick marks reflect the inferred position at which the endocranial characters discussed in this analysis transform. (1), sigmoidal endocast; (2), reduction of olfactory system; (3), shape of cerebrum in dorsal view [state 0 is an oval-shaped cerebrum; whereas state 1 is a rostrally tapering cerebrum]; (4), continuous dorsoventral height of the cerebrum; (5), anastomosis of internal carotid canals; (6), laterally displaced optic lobes; (7), rostrally expanded cerebellum; (8), absence of dural peak; and (9), folded cerebellum.

The general sigmoidal shape of the endocast in lateral view (Fig. 2) is a maniraptoran feature also present in ZPAL MgD-I/95 (Figs. 4, 5). It contrasts with the long, narrow endocranial cavity of non-coelurosaurian tetanurans, which reflects a plesiomorphic lack of relative forebrain inflation and a loose association between the brain and its enveloping dural sinuses (most significant in the mid- and hindbrain regions) [15], [16], [19], [29]–[31]. Orbit size has a significant effect on the shape of the brain in living birds [32], [33]; therefore, this derived shape also may reflect a paedomorphic retention of enlarged orbits within Maniraptora [33]. Retraction of the olfactory tracts and bulbs, so that they make up less than 0.5% of the total endocranial volume, is shared with ZPAL MgD-I/95, *Incisivosaurus* [34], and Aves (Figs. 4, 5) [20]. The same olfactory structures of deinonychosaurs (e.g., *Zanabazar junior* [35]) and the avialan *Archaeopteryx lithographica* [14], [20] exhibit some reduction when compared to non-maniraptoran coelurosaurs [15], [16], [19], [31] but still comprise approximately 5.0% of total endocranial volume (Fig. 4) [20]. A reduced olfactory system is thus a shared feature of maniraptorans; whereas, extreme volumetric reduction below ∼1.0% of total endocranial volume is a derived feature unique to oviraptorosaurs and Aves (Fig. 5). Outliers, however, are known among living birds. The black vulture, *Coragyps atratus*, and the albatross, *Phoebastria immutablis*, for example, have olfactory systems that make up approximately 2.7% and 1.4% of total endocranial volume, respectively [20].

The absence of a gradual rostral tapering of the lateral margin of the cerebrum, producing an oval-shape in dorsal view is shared with ZPAL MgD-I/95 [21] and with non-maniraptoran coelurosaurs (Figs. 4, 5). The contrasting pyriform morphology characterizes Aves, *Archaeopteryx*, *Zanabazar* and the basal oviraptorosaur *Incisivosaurus* (Fig. 4) [14], [34]–[35]. The polarization of this character with regards to oviraptorosaurs is therefore ambiguous. Either *Conchoraptor* contains a secondarily derived expression of the plesiomorphic coelurosaur condition or *Incisivosaurus* is autapomorphically convergent on the derived paravian condition (Fig. 5).

The cerebral hemispheres in both specimens of *Conchoraptor* extend beyond the lateral margin of the optic lobes. This condition was posited by Kundrát (2007) [21] as a character shared between *Conchoraptor* and the extant ostrich *Struthio camelus* to the exclusion of *Archaeopteryx* and thus a possible synapomorphy uniting oviraptorosaurs and Aves. The reconstruction of *Archaeopteryx* used by that study does in fact show this condition; although, it is likely due to the endocast being displayed at a slightly non-orthogonal angle [14]. We interpret the cerebrum in *Archaeopteryx* as extending laterally well beyond the edge of the optic lobes (Fig. 3C) [20]. This reinterpretation indicates that the *Conchoraptor* morphology is the conserved expression of the plesiomorphic coelurosaurian condition (Fig. 5) [15], [16], [19], [20], [29]–[31], [34]–[35].

The relatively constant depth of the cerebrum along its rostrocaudal length in IGM 100/3006 is also present in the therizinosaur *Erlikosaurus andrewsi* [19] but contrasts with the forebrain seen in tyrannosaurids, *Zanabazar*, *Archaeopteryx*, and Aves where the caudal region of the cerebrum expands dorsoventrally (Fig. 4) [14], [15], [20], [31], [35]. This expansion is exaggerated in paravians and partially accounts for the relatively large cerebral and total endocranial volumes. *Incisivosaurus* also had an expanded caudal forebrain, indicating that the disc-like morphology of *Conchoraptor* is a derived feature that evolved somewhere within the oviraptorosaur radiation (Fig. 5). This does not necessitate that the volumetrically reduced cerebrum of *Conchoraptor* is secondary. These values corroborate the hypothesis of Balanoff et al. (2013) [20] that the significant cerebral expansion characterizing modern birds began early in maniraptoran history (Fig. 5).

A subtle protuberance lies along the sagittal midline at the frontal-parietal suture of IGM 100/3006—the same position described as housing an epiphyseal projection in ZPAL MgD-I/95 [21]. The protuberance is mediolaterally expanded, reaching across the cerebrum and therefore more likely reflects the overlapping nature of the frontal-parietal suture rather than a pineal body [30]. All oviraptorosaurs, including the basally diverging *Incisiviosaurus*, exhibit relatively short frontals and extended parietals [4], . As noted by Kundrát (2007) [21] for ZPAL MgD-I/95, this condition places the frontal-parietal suture at approximately the rostrocaudal midpoint of the underlying cerebral hemispheres. This relationship is confirmed in IGM 100/3006 (Fig. 2) and *Incisivosaurus*, and we posit it as an oviraptorosaur synapomorphy (Fig. 5).

A pituitary body was not described for ZPAL MgD-I/95 [21]. This omission likely reflects the poor ossification of the surrounding basisphenoid that is a derived feature of Oviraptorosauria [4], [8]. The volume of the pituitary body relative to total endocranial volume is probably larger than indicated because of the failure to reconstruct the infundibular stalk. The reported relative volume, however, does fall within the range of living birds. The pituitary body in *Zanabazar* makes up over 1% of the total endocranial volume, whereas, that of *Alioramus* makes up approximately 2%. It has been suggested that pituitary volume is not phylogenetically variable but rather scales with body size [37]. The failure of the internal carotid arteries to anastomose, unlike the condition in Aves [38], [39], is a plesiomorphic condition also found in *Incisivosaurus* [34], *Zanabazar* [35], and *Archaeopteryx* (personal observation of Digimorph.org/specimens/*Archaeopteryx_lithographica*) (Fig. 5).

Lateral displacement of the optic lobes of IGM 100/3006 through expansion of the cerebrum and/or cerebellum is a derived condition shared with other maniraptorans, including *Incisivosaurus* [33] and paravians (e.g., *Zanabazar* [35]), *Archaeopteryx* [14], [20], and Aves [12]; Fig. 4). The relatively large size and spherical shape of the optic lobes in IGM 100/3006 compare closely with those of ZPAL MgD-I/95 and other maniraptorans (Fig. 4) [21] with the notable exception of *Incisivosaurus*. In *Incisivosaurus*, the optic lobes are more rectangular than spherical (Fig. 4) [34], perhaps reflecting the shape of the lobe or perhaps reflecting the influence of an adjacent structure such as a dural sinus. Either way, the condition in *Incisivosaurus* is interpreted as autapomorphic (Fig. 5).

The rostral expansion of the cerebellum in IGM 100/3006 compares closely with that of ZPAL MgD-I/95, *Incisivosaurus* and deinonychosaurs in retaining a small but distinct gap between the cerebellum and posterodorsal surface of the cerebrum. The gap is apomorphically closed in *Archaeopteryx* and Aves through overlap of the cerebellum onto the cerebrum [14], [40]. The cerebellum is apomorphically wide in IGM 100/3006 and ZPAL MgD-I/95, and based on comparisons within non-avialan theropods (e.g., *Majungasaurus* [30]), therizinosaurs [19], deinonychosaurs (e.g., *Zanabazar* [35]), and the basal oviraptorosaur *Incisivosaurus* [34] (Fig. 3), is the product of a character transformation inside Oviraptorosauria (Fig. 4). The loss of a distinct dural peak is a derived feature shared with ZPAL MgD-I/95, *Incisivosaurus*, and avialans. The peak is plesiomorphic for coelurosaurs [15], [16], [30], [31] and retained in the purported sister taxon to oviraptorosaurs, Therizinosauria [19], as well as in the deinonychosaurs *Zanabazar* [35] and *Tsaagan mangas* [41] (Fig. 4). Therefore, its absence in oviraptorosaurs and avialans is likely convergent (Fig. 5).

In contrast to IGM 100/3006, ZPAL MgD-I/95 is described as having a folded cerebellum (Fig. 2B, D) [21]. Though the cerebellum in Aves is consistently folded, that folding is not consistently reflected on the endocast, largely due to functionally related thickening of the overlying meninges (see [42] for discussion). Such variation might not be expected between IGM 100/3006 and ZPAL MgD-I/95 if those specimens indeed represent the same biological species. In contrast to ZPAL MgD-I/95, IGM 100/3006 is not fully mature skeletally as its braincase sutures are not completely fused. It is thus possible that the expression of cerebellar folding on the deep surface of the parietal and supraoccipital is a late-stage transformation in *Conchoraptor* not yet present in IGM 100/3006. Meningeal thickening and loss of neuroanatomical detail on the archosaur endocast, however, typically occur in later ontogenetic stages (see [44] for ontogenetic series of *Rhea americana* endocasts). This trajectory predicts that if there is ontogenetic disparity between the two *Conchoraptor* specimens it would be ZPAL MgD-I/95 that lacks the endocranial expression of cerebellar folding. It is also possible that the absence of a supraoccipital in ZPAL MgD-I/95 obfuscates the detail of its cerebellar structure.

The flocculus of IGM 100/3006 is relatively larger than that of any other observed maniraptoran, including ZPAL MgD-I/95 (Fig. 2). The flocculus also extends further caudally and has a more tapered distal end than that of ZPAL MgD-I/95, though the latter may have been truncated prematurely based on the shape of its reconstruction [21]. Although Kundrát (2007) [21] noted that the flocculus of *Struthio* has a more ventral orientation than that of either *Conchoraptor* or *Archaeopteryx*, the caudolateral orientation expressed in both specimens of *Conchoraptor* compares closely to that of known non-avian maniraptorans and falls well within the range of variation of modern birds (Fig. 4) [42], [43], [45]. This is the “Type 2” floccular morphology of Walsh et al. (2013) [45], which is characterized by an enclosed arterial loop, dome-shaped base, and rostrocaudal compression at its distal end (Fig. 2). The functional and/or ecological implications of a relatively large flocculus in *Conchoraptor* are unclear. No significant correlation exists between the volume of the floccular fossa and flight style in Aves and there is only a weak correlation between floccular volume and brachial index [45]. Other authors have suggested the flocculus plays an important role in gaze stabilization—coordinating eye movements with movements of the head, neck and body—and tends to be enlarged in taxa that rely on quick movements of the head and/or body [46]. The medulla oblongata of IGM 100/3006 does not differ substantially from that described for ZPAL MgD-I/95 and other maniraptorans [12], [21].

The shape of the inner ear in *Conchoraptor* as reconstructed from IGM 100/3006 does not differ appreciably from that of *Incisivosaurus* (Fig. 3) [34]. The caudal extension of the kidney-shaped rostral semicircular canal beyond the level of the common crus is found in all observed maniraptorans including *Incisivosaurus, Tsaagan*, *Archaeopteryx*, and Aves (Fig. 5) [14], [34], [41]. In more basal coelurosaurs such as tyrannosaurids, however, the caudal limit of the rostral semicircular canal ends at the common crus [15], [20]. Although the phylogenetic position of therizinosaurs is somewhat contentious [6]–[8], the inner ear morphology of *Erlikosaurus* is more consistent with a basal coelurosaur than a maniraptoran [19]. The ventromedial extension of the cochlear canal is widely distributed and plesiomorphic for theropods.

**Figure 6:**
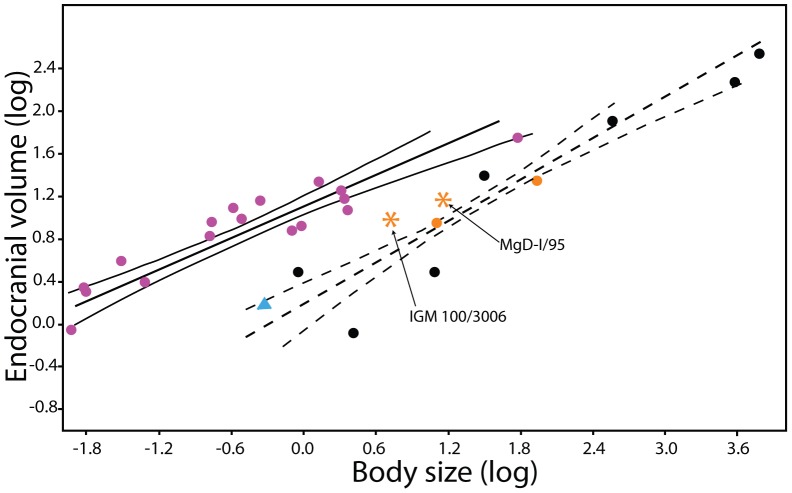
Log-log regression of total endocranial volume (cm^3^) and body mass (g). Modern birds are depicted as purple circles, non-avian dinosaurs as black circles, and *Archaeopteryx lithographica* (BMNH 37001) as a blue triangle. Specimens of *Conchoraptor gracilis* (IGM 100/3006 and MgD-I/95) are shown as asterisks. 95% confidence intervals are included for the reduced major axis regression lines through modern birds (solid) and non-avian dinosaurs (dashed). Although both specimens of *Conchoraptor* plot at the limit of the distribution of non-avian dinosaurs, they are well outside the distribution of living birds. Oviraptorosaurs (orange circles) are solidly within the distribution of non-avian dinosaurs.

The encephalization index of endocranial volume to body size places IGM 100/3006 and MgD-I/95 within the expected range of non-avian maniraptorans but outside that of crown-group birds (Fig. 6). This relative position contradicts the conclusion of Kundrát (2007: fig. 3) [21] that the encephalization of ZPAL MgD-I/95 was circumscribed by those of modern birds. Both specimens of *Conchoraptor gracilis*, however, do fall at the upper periphery of the distribution of non-avian dinosaurs (Fig. 5), whereas the other examined oviraptorids (i.e., *Citipati* IGM 100/978 and *Khaan* IGM 100/973) fall well within the distribution of non-avian dinosaurs (Fig. 5) [20]. The disparity in relative encephalization between the two specimens of *Conchoraptor* might be a reflection of three basic differences between our study and that of Kundrát (2007) [21]. These differences include: 1) we use femur length to approximate the body mass of *Conchoraptor* (5.25 kg) rather than using the body weight of the extant ratite *Rhea americana* as a proxy for this taxon (10–20 kg); 2) our comparative sample is expanded, especially for non-avian theropods including additional oviraptorosaurs; and 3) our endocranial volume for *Conchoraptor* of 9.44 cm^3^ is notably smaller than the 14.6 cm^3^ obtained by Kundrát (2007) [21] despite the fact that the sizes of the braincases are similar (Table 1). The reason for the marked volumetric disparity between the specimens of *Conchoraptor* is unclear because the braincases of the two specimens are highly comparable in size based on external measurements (Table 1). The difference may be partly explained by ontogenetic variation and the conservation of a developmental trajectory in which the brain develops faster than the surrounding braincase. The observed difference in encephalization seems much larger than the difference in skeletal maturity, and we suspect that the relative quality of the datasets may play a larger role here than biological variation. Log plots of forebrain volume versus body mass and forebrain volume versus total endocranial volume further demonstrate that IGM 100/3006 falls within the expected range of non-avian dinosaurs [20], and that a unique relationship between *Conchoraptor* and Aves to the exclusion of other non-avian theropods is not supported.

## Conclusions

The well-preserved nature of IGM 100/3006 allows us to build on observations of Kundrát (2007) and Kundrát and Janacek (2009) [21], [22] and provide a more complete picture of the endocranial morphology of *Conchoraptor gracilis* and its relationship to that of avialans. This picture includes a number of structures previously unknown for *Conchoraptor* including the pituitary body and the relationship among cranial nerves and vessels to the endocranial cavity. In the broad areas of morphological overlap, our observations and interpretations largely concur with those of Kundrát (2007) [21], though with some noteworthy differences. We cannot, for example, confirm the presence of a pineal body, nor did we observe any folding of the cerebellum (although distinct ridges are present on the cerebellar endocast of *Incisivosaurus* [34]). Increased sampling also revealed that many of the endocranial features promoted by Kundrát (2007) [21] as uniquely shared with *Conchoraptor* and Aves and evidence of an avialan origin for oviraptorosaurs, reflect the shared conservation of a plesiomorphic maniraptoran condition that is becoming increasingly “avian” (Fig. 5). This general pattern also is reflected in the encephalization indices, which place *Conchoraptor* among the most bird-like of the non-avian maniraptorans but still outside the observed range for Aves (Fig. 6). Not all of the features contributing to the endocranial similarity of *Conchoraptor* and Aves can be as easily explained. A number of these features are unique between the two taxa. And though we interpret them as convergence (e.g. homoplasy) based on the generally accepted phylogenetic pattern, we recognize that the evolution of the oviraptorosaur endocranial cavity may be of particular interest to avian neuroanatomists. The recognition that structures once considered unique to the avian brain evolved independently, or at least semi-independently, in a closely related lineage sets the stage for attaining a deeper understanding of the processes and constraints under which these features arise and become functionally integrated in the brain.

## References

[1] Gauthier JA (1986) Saurischian monophyly and the origin of birds. Mem Calif Acad of Sc 8:1–55.

[2] Maryańska T, Osmólska H, Wolsan M (2002) Avialan status for Oviraptorosauria. Acta Palaeontol Pol 47:97–116.

[3] Paul GS (2002) Dinosaurs of the Air: The Evolution and Loss of Flight in Dinosaurs and Birds. Baltimore: The Johns Hopkins University Press. 460 p.

[4] Osmólska H, Currie PJ, Barsbold R (2004) Oviraptorosauria. In: Weishampel DB, Dodson P, Osmólska Heditors. The Dinosauria, 2^nd^ Ed. Berkeley: University of California Press. pp.165–184.

[5] Senter P (2007) A new look at the phylogeny of Coelurosauria (Dinosauria: Theropoda). J Syst Palaeontol 5:429–463 10.1017/S1477201907002143

[6] Choiniere JN, Xu X, Clark JM, Forster CA, Guo Y, et al (2010) A basal alvarezsauroid theropod from the early late Jurassic of Xinjiang, China. Science 327:571–574 10.1126/science.1182143 20110503

[7] Zanno LE (2010) A taxonomic and phylogenetic re-evaluation of Therizinosauria (Dinosauria: Maniraptora). J Syst Palaeontol 8:503–543 10.1080/14772019.2010.488045

[8] Turner AH, Makovicky PJ, Norell MA (2012) A review of dromaeosaurid systematics and paravian phylogeny. Bulletin Am Mus Nat Hist 371:1–206 10.1206/748.1

[9] Xu X, You H, Du K, Han F (2012) An *Archaeopteryx*-like theropod from China and the origin of Avialae. Nature 475:465–470 10.1038/nature10288 21796204

[10] Jerison HJ (1969) Brain evolution and dinosaur brains. Am Nat 103:575–588.

[11] Jerison HJ (1973) Evolution of the Brain and Intelligence. New York: Academic Press. 482 p.

[12] Hopson JA (1979) Paleoneurology. In: Gans Ceditor. Biology of the Reptilia. NewYork: Academic Press. pp.39–146.

[13] Larsson HCE, Sereno PC, Wilson JA (2000) Forebrain enlargement among nonavian theropod dinosaurs. J Vert Paleontol 20:615–618.

[14] Alonso PD, Milner AC, Ketcham RA, Cookson MJ, Rowe TB (2004) The avian nature of the brain and inner ear of *Archaeopteryx* . Nature 430:666–669. 15295597 10.1038/nature02706

[15] Witmer LM, Ridgely RC (2009) New insights into the brain, braincase, and ear region of tyrannosaurs (Dinosauria, Theropoda), with implications for sensory organization and behavior. The Anatomical Record: Advances in Integrative Anatomy and Evolutionary Biology 292:1266–1296 10.1002/ar.20983 19711459

[16] Bever GS, Brusatte SL, Balanoff AM, Norell MA (2011) Variation, variability, and the origin of the avian endocranium: insights from the anatomy of *Alioramus altai* (Theropoda: Tyrannosauroidea). PLoS ONE 6:e23393 10.1371/journal.pone.0023393 21853125 PMC3154410

[17] Milner AC, Walsh SA (2008) Avian brain evolution: new data from Palaeogene birds (Lower Eocene) from England. Zool J Linn Soc-Lond 155:198–219 10.1111/j.1096-3642.2008.00443.x

[18] Walsh S, Milner A (2011) *Halcyornis toliapicus* (Aves: Lower Eocene, England) indicates advanced neuromorphology in Mesozoic Neornithes. J Syst Palaeontol 9:173–181 10.1080/14772019.2010.513703

[19] Lautenschlager S, Rayfield EJ, Altangerel P, Zanno LE, Witmer LM (2012) The endocranial anatomy of Therizinosauria and its implications for sensory and cognitive function. PLoS ONE 7:e52289 10.1371/journal.pone.0052289.s005 23284972 PMC3526574

[20] Balanoff AM, Bever GS, Rowe TB, Norell MA (2013) Evolutionary origins of the avian brain. Nature 501:93–96 10.1038/nature12424 23903660

[21] Kundrát M (2007) Avian-like attributes of a virtual brain model of the oviraptorid theropod *Conchoraptor gracilis* . Naturwissenschaften 94:499–504 10.1007/s00114-007-0219-1 17277940

[22] Kundrát M, Janáček J (2007) Cranial pneumatization and auditory perceptions of the oviraptorid dinosaur *Conchoraptor gracilis* (Theropoda, Maniraptora) from the Late Cretaceous of Mongolia. Naturwissenschaften 94:769–778 10.1007/s00114-007-0258-7 17530209

[23] Osmólska H (2004) Brief report: Evidence on relation of brain to endocranial cavity in oviraptorid dinosaurs. Acta Palaeontol Pol 49:321–324.

[24] Iwaniuk AN, Nelson JE (2002) Can endocranial volume be used as an estimate of brain size in birds? Can J Zool 80:16–23 10.1139/z01-204

[25] Witmer LM, Ridgely RC, Dufeau DL, Semones MC (2008) Using CT to peer into the past: 3D visualization of the brain and ear regions of birds, crocodiles, and nonavian dinosaurs. In: Endo H, Frey Reditors. Anatomical Imaging: Towards a New Morphology. Tokyo: Springer Japan. pp.67–87.

[26] Christiansen P, Fariña RA (2004) Mass prediction in theropod dinosaurs. Hist Biol 16:85–92 10.1080/08912960412331284313

[27] Campione NE, Evans DC (2012) A universal scaling relationship between body mass and proximal limb bone dimensions in quadrupedal terrestrial tetrapods. BMC Biol 10:60 10.1186/1741-7007-10-60 22781121 PMC3403949

[28] Felsenstein J (1985) Phylogenies and the comparative method. Am Nat 125:1–15.

[29] Franzosa J, Rowe T (2005) Cranial endocast of the Cretaceous theropod dinosaur *Acrocanthosaurus atokensis* . J Vert Paleontol 25:859–864.

[30] Sampson SD, Witmer LM (2007) Craniofacial anatomy of *Majungasaurus crenatissimus* (Theropoda: Abelisauridae) from the Late Cretaceous of Madagascar. J Vert Paleontol 27:32–104 10.1671/0272-4634(2007)27 [32: CAOMCT]2.0.CO; 2

[31] Bever GS, Brusatte SL, Carr TD, Xu X, Balanoff AM, et al (2013) The braincase anatomy of the Late Cretaceous dinosaur *Alioramus* (Theropoda: Tyrannosauroidea). Bull Am Mus Nat Hist 376:1–72 10.1206/810.1

[32] Kawabe S, Shimokawa T, Miki H, Matsuda S, Endo H (2013) Variation in avian brain shape: relationship with size and orbital shape. J Anat 223:495–508. 24020351 10.1111/joa.12109 PMC4399353

[33] Bhullar B-AS, Marugán-Lobón J, Racimo F, Bever GS, Rowe TB, et al (2012) Birds have paedomorphic dinosaur skulls. Nature 487:223–226. 22722850 10.1038/nature11146

[34] Balanoff AM, Xu X, Kobayashi Y, Matsufune Y, Norell MA (2009) Cranial osteology of the theropod dinosaur *Incisivosaurus gauthieri* (Theropoda: Oviraptorosauria). Am Mus Nov 3651:1–35.

[35] Norell MA, Makovicky PJ, Bever GS, Balanoff AM (2009) A review of the Mongolian Cretaceous dinosaur *Saurornithoides* (Troodontidae: Theropoda). Am Mus Nov 3654:1–63.

[36] Xu X, Cheng Y-N, Wang X, Chang C-H (2002) An unusual oviraptorosaurian dinosaur from China. Nature 419:291–293 10.1029/2001 12239565

[37] Edinger T (1942) The pituitary body in giant animals fossil and living: a survey and a suggestion. Q Rev Biol 17:31–45.

[38] Baumel JJ, Gerchman L (1968) The avian intercarotid anastomosis and its homologue in other vertebrates. Am J Anat 122:1–18. 5654502 10.1002/aja.1001220102

[39] Aslan K, Atalgin H, Kürtül I, Bozkurt EU (2006) Patterns of the internal and cerebral carotid arteries in various avian species: a comparative study. Revue de médecine vétérinaire 157:619.

[40] Larsell O (1967) The Comparative Anatomy and Histology of the Cerebellum from Myxinoids through Birds. Minneapolis: University of Minnesota Press.

[41] Norell MA, Clark JM, Turner AH, Makovicky PJ, Barsbold R, et al (2006) A new dromaeosaurid theropod from Ukhaa Tolgod (Ömnögov, Mongolia). Am Mus Nov 3545:1–51.

[42] Ksepka DT, Balanoff AM, Walsh S, Revan A, Ho A (2012) Evolution of the brain and sensory organs in Sphenisciformes: new data from the stem penguin *Paraptenodytes antarcticus* . Zool J Linn Soc-Lond 166:202–219 10.1111/j.1096-3642.2012.00835.x

[43] Smith NA, Clarke JA (2012) Endocranial anatomy of the Charadriiformes: sensory system variation and the evolution of wing-propelled diving. PLoS ONE 7:e49584 10.1371/journal.pone.0049584.s003 23209585 PMC3507831

[44] Picasso MBJ, Tambussi CP, Degrange FJ (2010) Virtual reconstructions of the endocranial cavity of *Rhea americana* (Aves, Palaeognathae): postnatal anatomical changes. Brain Behav Evolut 76:176–184. 10.1159/000321173 21042004

[45] Walsh SA, Iwaniuk AN, Knoll MA, Bourdon E, Barrett PM, et al (2013) Avian cerebellar floccular fossa size is not a proxy for flying ability in birds. PLoS ONE 8:e67176 10.1371/journal.pone.0067176.s002 23825638 PMC3692442

[46] Witmer LM, Chatterjee S, Franzosa J, Rowe T (2003) Neuroanatomy of flying reptiles and implications for flight, posture and behaviour. Nature 425:950–953 10.1029/1999JC000190 14586467

